# A Unique Case of Foreign Body Acquired by Stabbing and Retained for 7 Years in the Sigmoid Colon

**DOI:** 10.3390/reports6020024

**Published:** 2023-05-24

**Authors:** Iulia Cristina Pîrvulescu, Alfred Najm, Eduard Cristian Popa, Alexandru Laurentiu Chiotoroiu, Sanda Maria Cretoiu, Bogdan Severus Gaspar

**Affiliations:** 1 Surgery Clinic, Emergency Clinical Hospital, 014461 Bucharest, Romania; 2 Department of Surgery, Carol Davila University of Medicine and Pharmacy, 050474 Bucharest, Romania; 3 Department of Morphological Sciences, Cell and Molecular Biology and Histology, Carol Davila University of Medicine and Pharmacy, 050474 Bucharest, Romania

**Keywords:** foreign body, glass blade, sigmoid colon, surgery, large bowel

## Abstract

The ingestion of foreign bodies is a common cause for presentation in the emergency department by pediatric, adult, or elderly psychiatric patients. Swallowed foreign bodies sometimes represent a great challenge for surgeons due to the obstruction or perforation of the digestive tube’s upper or lower segments. Occasionally, the foreign bodies detected in the lower parts of the digestive tube (colon and rectum) could be introduced through the anal route with the risk of perforation of the rectum or sigmoid colon. In this report, we describe a unique case of a foreign body located in the sigmoid colon, where it arrived due to backstabbing and was retained for 7 years without acute symptoms. The 43-year-old male patient came to the emergency department with pain in the left iliac fossa. Before his presentation, a computerized tomography (CT) scan examination had suggested a foreign body. A surgical approach was decided. The surgery started as an exploratory laparoscopy and was converted to a xiphoid-pubic incision to extract the foreign body (a piece of glass about 8 cm long) through a sigmoid colotomy followed by a double-layer sigmoidorrhaphy. The postoperative evolution of the patient was uneventful. As far as we know, this is the first case of a patient with a foreign glass body positioned in the sigmoid colon that got there by stabbing and not by ingestion or introduced per anum. In conclusion, we suggest that aggressive behavior and abdominal wall penetration by different sharp objects should be considered when foreign bodies are detected in the abdomen.

## 1. Introduction

Swallowing a foreign body is among the most common reasons for presentation in the emergency department. Depending on the localization, foreign body presence in the digestive tract may be completely asymptomatic or generate variable, nonspecific symptoms. In the majority of cases, foreign bodies may be accidentally swallowed, as in the case of children or the elderly, or maybe swallowed voluntarily, as in the case of those with alcohol intoxications, psychiatric patients, drug addicts, and prisoners [1]. The ingested structures include coins, stones, some small animal bones, including fish bones, metals and aluminum (batteries), graphite pencils, teeth, razor blades, and belts [2]. The complications can be severe and sometimes life-threatening depending on the swallowed object, its shape, and the time that passes until treatment [3]. Due to the high frequency of these cases, one can find in the literature guidelines on the best approaches concerning ingested foreign bodies [4,5]. The management of foreign body ingestion depends on the age and clinical condition of the patient. Other influencing factors should be considered: (1) the anatomic location of the object; (2) the classification of the swallowed material depending on the size, shape, and consistency; and (3) the necessity of an urgent intervention due to possible acute complications [6].

Patients identified with a foreign object in the proximal or distal gastrointestinal tract display signs and symptoms of transit blockage or perforation [7]. Surgical evaluation should be considered in case of symptoms such as acute dysphagia, fever, vomiting, signs of peritoneal irritation, hematemesis, or melena [8]. In such cases, the treatment of choice is surgery, normally needed in only 1–2.7% of the patients [9]. Foreign bodies can pass spontaneously in 80–90% of the cases or become blocked at several levels of the digestive tract, such as the esophagus (most commonly), the stomach, the small or large intestine, and the rectum [5]. The most common location is in the upper part of the digestive tube, and approximately 10–20% of the cases require the endoscopic extraction of these foreign bodies [10,11]. The diagnosis is based on anamnestic data, clinical examination, and laboratory tests and is supported by the radiological examination of various incidences, endoscopy and/or colonoscopy, and computer tomography (CT) examination. Radiography is the main examination used for evaluating these patients. In most cases, the foreign bodies found in the digestive tract appear as radiopaque on X-ray examination [10]. Herein, we report the case of a patient with a foreign body of glass lodged in the sigmoid colon for seven years. This case’s uniqueness is that the foreign body was not swallowed, but got into the colon by stabbing and penetrating the soft tissues to reach the colonic lumen. The glass blade was surgically removed in our surgery clinic, with a good postoperative outcome.

## 2. Case Presentation 

A 43-year-old male patient presented to the emergency room for pain in the left iliac fossa and the presence of a foreign body (confirmed to be a knife blade) discovered at a CT scan examination performed before his presentation. The patient had no significant medical-surgical history (according to him), but stated that he was the victim of an aggression that occurred about 7 years ago. The patient declared he suffered a backstabbing with a knife in an altercation after excessive alcohol consumption. The patient reported a febrile episode lasting several days post-aggression, without mentioning intestinal transit disorders at that time or any other symptoms in the last 7 years.

Clinical examination showed a conscious, cooperating, tempo-spatially oriented patient, in good general condition, afebrile, and hemodynamically and respiratory stable, with a 3 cm hypertrophic scar in the left sacral region, without other significant signs in the inspection of segments of the trunk and abdomen. The objective examination was continued with the palpation of the abdomen, which resulted in the appearance of pain upon palpation in the left iliac fossa but without any signs of peritoneal irritation.

**Figure 1:**
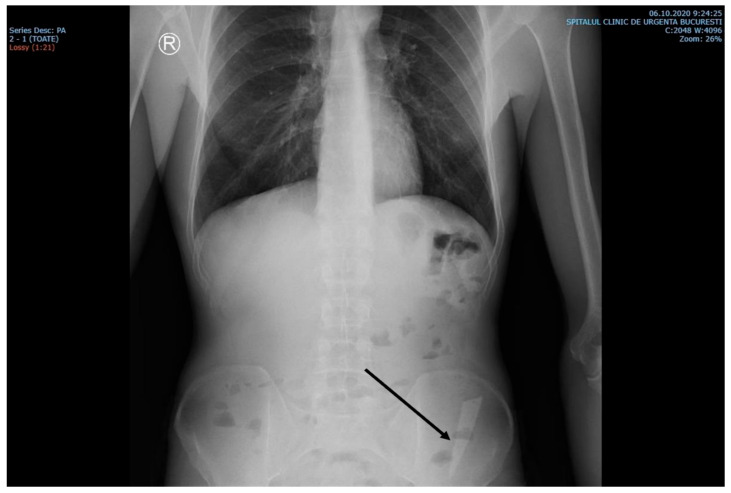
X-ray examination showed an elongated blade (arrow) in the iliac fossa, possibly in the sigmoid colon.

The laboratory examinations of the patient showed leukocytes = 9830/μL, hemoglobin = 12.8 g/dL, platelets = 379,000/μL, ALT = 69 U/L, AST = 48 U/L, PT = 13.6 s, and the rest within normal limits. A chest X-ray at admission showed no acute pleuro-pulmonary lesions. An abdominal X-ray at admission showed discrete aerocolia without radiologically noticeable pneumoperitoneum, hydroaeric levels, and a radiopaque structure in the left flank, denoting a foreign body (Figure 1).

**Figure 2:**
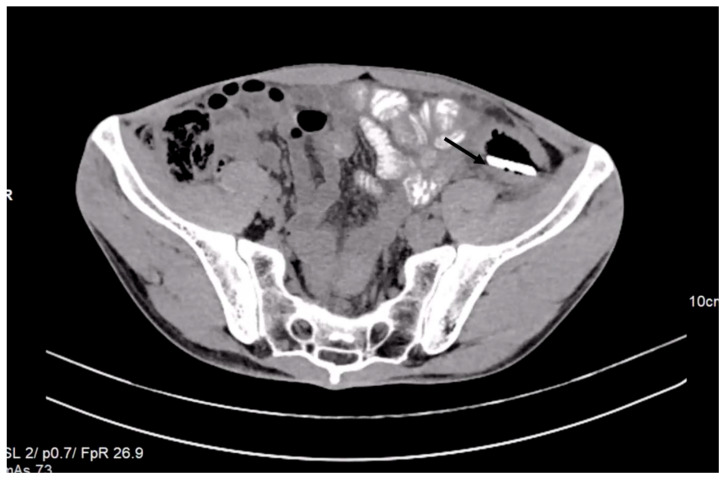
Contrast-enhanced abdominal CT revealed the presence of a foreign object in the left iliac fossa.

A CT scan performed before admittance showed, in the left iliac fossa, a foreign body with a density similar to the bone cortex, without reaching the specific density of the metal, and with an elongated shape similar to a knife blade tip (dimensions: 7.5 cm long at the base, 2 cm wide, and 4 mm thick). Most of this foreign body was located in the descending colon at the junction with the sigmoid. The tip of the foreign body was oriented craniocaudally and ventrally. It crossed the digestive wall and reached a transperitoneal position in the right abdominal muscle, on the left side, passing tangentially to the left lower epigastric vessels. In this region, the colonic wall appeared moderately thickened. There were small local intraperitoneal pericolic gas bubbles, no local fluid collections, no ascites, no pneumoperitoneum, and no signs of occlusion (Figure 2).

Corroborating all the data, the diagnosis of an intracolonic foreign body was established, and hydroelectrolytic rebalancing treatment was instituted, as well as an analgesic, gastric antisecretory, antibioprophylaxis, deep vein thrombosis prophylaxis, and colon preparation for surgery with an osmotic laxative containing a high molecular weight molecule—macrogol. It was decided to perform an exploratory laparoscopy, obtaining pneumoperitoneum by the Hasson method under general anesthesia. The exploratory laparoscopy was needed since the foreign body did not match the specific density of the metal (the patient declared he was stabbed with a knife), and also because the CT scan did not show any signs of fluid collection and the fact that the blade was shown to be passing tangentially to blood vessels.

After introducing the optical trocar at the supraumbilical level, adhesion syndrome was discovered in the left iliac fossa, consisting of the large omentum, sigmoid colon, and enteral loop; all other organs were of normal macroscopic appearance. Two working trocars were inserted into the right iliac fossa (10 mm) and suprapubic fossa (5 mm). Viscerolysis was performed by discovering a parietal abscess at the level of the left iliac fossa, which was evacuated and for which intense lavage was performed. The conversion was decided, and a xiphoid-pubic incision was performed. Upon the manual palpation of the descending colon, the foreign body was detected digitally, having a hard consistency located in the intraluminal axis of the colon.

**Figure 3:**
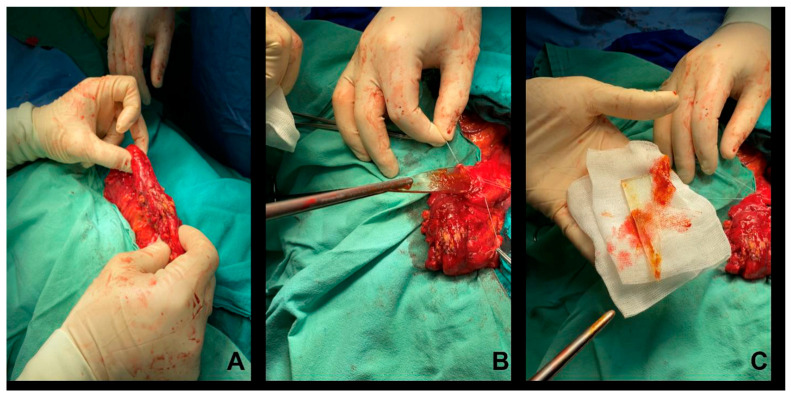
Macroscopic intraoperative picture of the process of extraction of the glass blade. (**A**) Intraoperative aspect of the colon containing the glass fragment. (**B**) Intraoperative aspect of the moment of extracting the fragment from the colonic lumen. (**C**) The glass fragment was extracted from the colon.

Next, a sigmoid colotomy of about 4 cm was decided and performed, and the foreign body was extracted—a piece of glass about 8 cm long, with a sharp end and a blunt one. (Figure 3). 

Intraluminal exploration was continued without any other detectable lesions. The patient’s vitals were monitored in the postanesthetic period, which was approximately 16 h. The patient was monitored for postoperative symptoms such as pain, nausea, vomiting, and antagonistic treatment of residual effects of anesthesia. The patient had an average BP of 107/74 mmHg, average heart rate of 63 bpm, temperature of 36.7 °C, average SaO_2_ of 96% without additional addition of O_2_, and diuresis of approximately 2400 mL of normochromic urine; the amount of gastric aspirate on the nasogastric tube was zero, peritoneal drainage showed no signs of bleeding, and the wound was normal. The patient received hydroelectrolytic, analgesic, anti-inflammatory, gastric antisecretory, antibiotic, and antiemetic treatment, as well as prophylaxis for deep vein thrombosis.

The nasogastric tube was postoperatively removed after 27 h, and the patient was moved out of the intensive care unit. He continued the prescribed treatment, and oral water intake was instituted. The patient resumed intestinal transit for gas and feces; digestive tolerance was good, and parietal-colic drainage was minimal. After five days, the last drainage tube was suppressed. The patient was reintroduced to solid food and continued the intestinal transit for feces. The patient was discharged in good general condition without other special mentions. The patient returned for control and removal of the sutures according to the instructions. He was monitored after one month by colonoscopy, displaying the normal aspect of the colon.

## 3. Discussion

Usually, foreign bodies found in the digestive tract are a common issue in the emergency department. Foreign bodies in the sigmoid colon also are quite frequent findings in the clinical practice of a surgeon due to accidental ingestion, especially in young children or elders [12,13,14]. The majority of foreign bodies are ingested and pass through the digestive tube without any complications in 80-90% of the cases [15,16]. 

This paper presents the unique and peculiar case of a patient with a foreign body of glass detected in the descending/sigmoid colon in our general surgery clinic of the Emergency Clinical Hospital of Bucharest.

The diagnosis of an ingested foreign body might be easily overlooked in those patients where anamnesis fails to identify an adequate history for swallowed objects or in cases where the foreign body is not seen through classical imagistic techniques [17].

To our knowledge, there is no case in the literature that describes a foreign body in the colon that arrived there by external introduction after aggression. Foreign bodies can reach the colon by swallowing or entering through the anus and migrating proximally [18]. 

The symptomatology of a foreign body present in the intestines is not pathognomonic. Signs and symptoms are very diverse, consisting of nausea, vomiting, anorexia, intermittent spasmodic abdominal pain, acute or chronic abdominal pain, and bloody stools, accompanied or not by fever or by signs of acute perforation [19,20]. If the retention is prolonged, one might even encounter weight loss [21]. 

In the present study, the symptomatology consisted only of chronic pain in the left iliac fossa, absolutely nonindicative of the presence of a foreign body. 

The possible increase in the number of cases and the number of different signs and symptoms, from a “silent perforation” with minimal symptoms to acute abdomen, brings into discussion the need to establish guidelines for managing ingested foreign bodies within the colon. The literature describes management protocols for upper and lower foreign objects discovered in the gastrointestinal tube [1,22,23]. In general, the management of these cases depends on the size, shape, material, and location of the foreign object, and the surgical treatment (necessary in 1% of the cases) is indicated for the occurrence of complications [24]. 

Regardless of the location of the foreign body, in the case of sharp and elongated objects, such as fish bones, chicken bones, pieces of glass, and toothpicks, there is a risk of intestinal obstruction or perforation [25,26]. Generally, the perforation is followed by bleeding or the formation of an abscess or fistula after a long retention time [21]. 

A large-dimension glass blade in the colon is a unique case and can be life-threatening. It also represents an unusual situation for the patient and the surgeon. There are no specific guidelines for such rare cases; however, one cannot imagine that in such a case as ours, there was no perforation. A “silent perforation” must also be suspected, because sometimes, the perforation does not manifest through acute clinical signs [27].

Multiple cases of acute or chronic intestinal perforation by a foreign body are described in the literature. Acute perforation is more likely to be observed in the small intestine, while chronic perforation occurs more often in the sigmoid colon [28]. 

Foreign bodies in the sigmoid colon have various ways of presenting to the emergency department. One must remember how important it is to obtain as much information about the way the foreign body entered the gastrointestinal tract and rely on CT and colonoscopy, when possible, to detect the position and if a perforation occurred. Large bowel perforation by foreign bodies is a surgical emergency due to a high mortality rate, and it has been described with a frequency of 20.3% in the colon, 5.5% in the sigmoid, and 10.6% in the rectum [29].

In rare circumstances, acute abdomen can occur due to sigmoid colon perforation. Such a case was described by Alonso-Gómez et al. of a 35-year-old schizophrenic man who ingested four foreign bodies (two batteries and two needles), one needle perforating the colon. Although the object was small, the removal required surgical intervention [30]. In this case, due to the lack of intra-abdominal contamination and the characteristics of the lesion (punctate perforation and lack of fecal material around the perforation), a primary suture was performed, as opposed to our case, where the surgical techniques and approach were complex due to the size and shape of the foreign object. 

In such cases, a CT scan usually reveals a pneumoperitoneum’s presence. Our patient did not present pneumoperitoneum. A CT scan examination revealed local intraperitoneal pericolic gas bubbles and no pneumoperitoneum, which was evidence for chronic perforation. 

In connection with chronic perforation, Ali et al. described the case of a mentally retarded boy who retained a flat piece of plastic in the sigmoid colon for more than a year, also raising the problem of the way it got into that position, either by ingestion or by anal introduction [31]. 

This case is similar to the case presented here regarding the long period of retention in the colon and because of chronic perforation discovered at the moment of surgical laparotomy. In our case, the peculiarity was that the glass blade was lodged for 7 years in the sigmoid colon after a backstabbing of the patient. In the present case, chronic perforation was suspected based on the CT scan description of a foreign body with a sharp end penetrating the intestinal wall into the right abdominal muscle. CT scan imaging is able to predict with 82% to 90% accuracy if there is a large intestine perforation [32]. 

Moreover, the colonic wall appeared moderately thickened at the place of penetration. We were not certain what kind of foreign object was present in the sigmoid colon, because the X-ray and CT density of the object did not match the patient’s declarations, declaring he was stabbed with a knife blade. Generally, glass pieces appear on the radiography as radiopaque structures, being easy to visualize if the radiography is performed using the appropriate incidence [33]. Viewing a piece of glass on an X-ray depends on the size of the piece, but the density of the glass, regardless of size, is generally sufficient to differentiate a piece of glass from the surrounding tissues [34], as it was in our case.

In specific cases, the foreign body can be extracted from the lower segments of the alimentary canal through colonoscopy. Ma et al. described the colonoscopic extraction of an ingested jujube pit from a 78-year-old man with sigmoid colon perforation [35].

In the case of a small foreign body, sometimes it can become embedded in the wall of the intestine, as in a case described by Tierney et al., where a 4 cm linear bone fragment was removed by sigmoidoscopy with rat-tooth forceps [36]. 

In another interesting case of a foreign body lodged in both walls of the sigmoid colon, a toothpick was removed by flexible sigmoidoscopy [19].

There are circumstances when a swallowed object might cause chronic gastrointestinal symptoms. Muller et al. described the case of a pediatric patient with symptoms that mimicked Crohn’s disease, supporting the idea that foreign bodies in the colon result from swallowing and might have a chronic evolution and subacute obstruction. This case was diagnosed through colonoscopy for the foreign object (a ball pen cap) embedded in the sigmoid mucosa [21]. The object was not removed due to the risk of perforation, but passed spontaneously, being an example of minimally invasive treatment. Due to the large dimensions of the foreign body revealed in the sigmoid colon of our patient by CT scan imaging, we decided to perform an exploratory laparoscopy to visualize the peritoneal cavity and the relationship of the foreign body with the vascular structures. In the current case, there was also the theoretical assumption that maybe the foreign body was in the peritoneal cavity, since there had been no signs of intestinal obstruction for seven years, even though the possibility was very low. Although an imagistic investigation did not reveal any collection, we identified a parietal abscess at the level of the left iliac fossa, which was evacuated, and we converted the laparoscopic intervention to open surgery.

## 4. Conclusions

In conclusion, we present here a rare case of a colonic foreign body. The foreign body was a piece of glass shaped like a knife blade that did not get into the colon by ingestion, but by backstabbing during an altercation. The piece of glass passed through the soft tissues and migrated to the colonic lumen and was retained for 7 years without symptoms. When encountering a similar case, it is important to have as much information about the event, the type of penetrating object (its length, width, and structure), the direction of the attack, and if the stomach and the urinary bladder were full or not. A complete detailed anamnesis has to be performed, because such injuries can be followed by severe complications, such as life-threatening bleeding, acute or chronic perforation, damage of the major intra-abdominal organs, and sepsis. The clinical examination has to be performed repeatedly by the same experienced surgeon to remark on a change in the status of the patient while the patient is imagistically investigated. The surgeon will decide the best approach with the best possible outcome for the patient. A minimally invasive procedure (laparoscopy) is useful for the patient, but it is important to decide at the right time to make the conversion to open surgery.

Frequently, cases of ingestion of foreign bodies can be found in the specialized literature. Still, none describes a piece of glass that penetrated the lumen of the colon by stabbing, this being the peculiarity of the current case.

## Data Availability

Not applicable.
